# His Majesty's Visit. Many Difficulties Overcome

**Published:** 1917-09-08

**Authors:** 


### THE LONDON HOSPITAL.

### His Majesty's Visit. Many Difficulties Overcome.

SINCE the last meeting of the Court in June the hospital's work has been as heavy as ever, and every quarter it becomes more and more difficult to secure an adequate staff, more difficult to obtain supplies. And yet, happily, the difficulties have never been insurmountable, and the hospital continues to "carry on."

MANY DIFFICULTIES OVERCOME.

The committee thank the various bodies concerned for the sympathetic way in which the hospital has been treated. When the hospital authorities have been in despair of obtaining house physicians and house surgeons, the War Office has helped by lending men for periods of three months; lady doctors also have given service, and doctors have come from America to help.

When there has been trouble as to the supply of articles which come under the head of "building material"—screws, timber, slates, steel, etc.—the hospital has always received courteous assistance from the Ministry of Munitions: with regard to the lay staff, the engineers, electricians, etc., the views of the committee have been always sympathetically and kindly heard by the local Tribunal: when trouble was threatened with the coal supply, the great coal merchants put their information and advice at the disposal of the hospital. The assistance received over and over again from such great societies as the Red Cross and Queen Mary's Needlework Guild and many others, which have sent hundreds of pounds' worth of dressings and ward necessities, are deeply appreciated. When there was a difficulty owing to the shortness of taxi-cabs in getting the staff down quickly from the West End at night to do important operations, a gentleman at once came to the assistance of the hospital by putting his car at its disposal for the period of the war, and there is hardly a night but this car goes West to fetch a surgeon to save some poor East-Ender's life. These many and varied kindnesses have taught the committee that difficulties disappear when attacked: a source of hopefulness for the future.

Votes of sympathy have been tendered by the committee to Mr. John Henry Buxton, one of the staunchest of the hospital's friends, on the death of his son, Mr. Andrew Buxton, at the Front; and to Sir Evan Spicer, who received a telegram that his son had been killed, fortunately corrected later to "seriously wounded."

DAYLIGHT AIR-RAIDS.

Since the last quarterly Court the London Hospital has had the new experience of helping in respect of day air-raids. That of June 13 came without any warning at all, and was a very terrible experience for the East End. No fewer than 207 patients arrived at the main entrance for treatment within an hour. Most of these could be sent to their own homes after receiving first-aid treatment, but seventy-five were so seriously hurt that they had to be admitted to the wards; forty-four died. Mr. Wynne Baxter held inquests at the hospital on these victims, and the jury sent the following communication to the hospital authorities: "In the opinion of the jury the conduct of the staff of the London Hospital under the trying circumstances of the recent air-raid is deserving of the highest commendation."

The committee were impressed with the splendid behaviour of the police and of the drivers and conductresses of the 'buses on this occasion, and also at the time of the raid on July 7; and letters of thanks and appreciation were sent to Sir Edward Henry, Chief of Police, and to the secretary of the General Omnibus Company. One of the most distressing features is the visits of relatives on the evening of the day of the raid. The first intimation many people have of the injury to their dear ones is the non-arrival home from work at the end of the day, and then a dreadful search of the hospitals begins.

With regard to these daylight raids, the committee have done all that they can to protect the patients within the hospital, and to organise the admission of wounded.

VISIT OF H.M. THE KING.

His Majesty the King visited the hospital in the afternoon of the raid of June 13, and with characteristic kindness spoke to the injured and sympathised with them. He left for Scotland the same day, and wired that evening asking how the patients were progressing.

THE VENEREAL DEPARTMENT.

The working of the Venereal Department still progresses, although Mr. Frank Kidd, head of one of its departments, has been called up and is now somewhere in France.

It will be remembered that under the agreement with the authorities the hospital, for the sum of £3,000, has to

(1) Treat all patients suffering from venereal disease, whatever their station in life, who may ask to be treated. No payment is to be made. Strict secrecy is to be maintained, and every session must be held to suit the convenience of patients.

(2) Examine specimens sent to the hospital by doctors, and give a report as to whether the patient is or is not suffering from venereal disease.

(3) Give doctors full and free facilities for learning how to treat the disease and how to inject salvarsan.

(4) Provide salvarsan free of charge to approved outside doctors if patients prefer to be treated by their own doctors.

Since the last Court three assistant surgeons have been called up, Mr. Frank Kidd, Mr. Warren, and Mr. Milne. This has necessitated a complete re-allotment of surgical beds throughout the hospital, and all members of the staff remaining are doing extra work. It is impossible, however, to admit any but urgent cases.

HONOURS FOR THE STAFF.

The Court will be glad to hear that the hospital has been well represented in the new Orders of Chivalry. The matron, Miss Lückes, has been made a Companion of the British Empire. The Hon. W. H. Goschen and Sir Douglas Owen, both members of the committee, have been created Knights of the British Empire. Lord Burnham, another member of committee, has been made a Companion of Honour.

Mr. Walter Marlborough Pryor, a member of the committee (now Major Pryor), has been awarded the D.S.O.

The assistant secretary, Mr. Arthur Elliott, now a lieutenant in the Grenadier Guards, has been slightly wounded—shot through the arm—and his many friends are all glad to know of his rapid convalescence.

The Grant from the Metropolitan Hospital Sunday Fund for this year amounted to £8,065 9s. 4d., being nearly £1,000 more than the previous year.

